# Malnutrition, anaemia and anisocytosis as public health problems among children ≤ 5 years living in malaria perennial transmission areas of Mount Cameroon: a cross sectional study

**DOI:** 10.1186/s41182-022-00469-6

**Published:** 2022-10-24

**Authors:** Rene Ning Teh, Irene Ule Ngole Sumbele, Gillian Asoba Nkeudem, Sorelle Mekachie Sandie, Sharon Odmia Sama, Samuel Metuge, Helen Kuokuo Kimbi

**Affiliations:** 1 Department of Zoology and Animal Physiology, University of Buea, Buea, Cameroon; 2 Department of Social Economy and Family Management, Higher Technical Teachers’ Training College, University of Buea, Kumba, Cameroon; 3 3Deprtment of Health Sciences, Biaka University Institute, Buea, Cameroon; 4 Department of Medical Biomedical Sciences, The University of Bamenda, Bamenda, Cameroon; 5 Centre for Molecular Parasitology, Department of Microbiology and Immunology, Drexel University College of Medicine, Philadelphia, USA

**Keywords:** *Plasmodium*, Microcytic anaemia, Anaemia, Anisocytosis, Stunting, Children ≤ 60 months

## Abstract

**Background:**

Anaemia, anisocytosis, malnutrition (especially stunting) are common health problems in developing countries with children being the most vulnerable. These conditions have negative impacts on human performance, growth and development, and can further be complicated if comorbidity exists within a holoendemic stratum with strong and perennial malaria parasite transmission such as the Mount Cameroon area. The study aimed at determining the prevalence and severity malnutrition, anaemia and anisocytosis in children ≤ 5 years, living in the conflict hit malaria perennial transmission zone of the Mount Cameroon area.

**Method:**

A cross-sectional community-based survey involving 628 children ≤ 5 years was conducted. Malaria parasitaemia was confirmed by Giemsa-stained microscopy and the density was log transformed. Haemoglobin (Hb), mean cell volume and red blood cell distribution width were estimated using an auto-haematology analyser and defined according to WHO standards. Anthropometric indices were analysed and compared with WHO growth reference standards using WHO Anthro software.

**Results:**

*Plasmodium* infection, anaemia, microcytic anaemia, anisocytosis and stunting were prevalent in 36.0, 72.8, 30.1, 54.1 and 29.0% of the children, respectively. The ≤ 24 months children were more moderately stunted (14.7%), with higher prevalence of microcytic anaemia (38.8%) and anisocytosis (68.8%) (*P* < 0.002 and *P* < 0.001, respectively) when compared with the older children. The mean Hb level in the study population was 10.04 g/dL with children ≤ 24 months having the least mean haemoglobin level (9.69 g/dL) when compared with their older counterparts at *P* < 0.001. The odds of having anisocytosis were highest among children who were malnourished (OR = 4.66, *P* = 0.005), those infected with malaria parasites (OR = 1.85, *P* = 0.007), and whose parents had a primary (OR = 3.51, *P* = 0.002) and secondary levels of education (OR = 2.69, *P* = 0.017).

**Conclusion:**

Malaria, anaemia, anisocytosis and undernutrition still remain severe public health concerns among children ≤ 60 months in the Mount Cameroon area. This therefore emphasizes the need for the implementation of consistent policies, programmes and activities to avoid malaria, anaemia, anisocytosis and stunting in the paediatric age group.

## Background

The World Health Organization (WHO) African Region continues to bear the greatest burden of malaria, with children under the age of five bearing the greatest toll. Globally, the region accounted for 95% of all malaria cases (228 million) and 96% of all malaria deaths (602 000) in 2020, with Cameroon accounting for 2.9% and 2.4% of these numbers, respectively[1]. Between 2012 and 2018, 4,052,216 cases of malaria were diagnosed in children under 5 years of age in Cameroon, either by microscopy or RDT, with a progressive increase per year from 369,178 in 2012 to 652,661 in 2018 [2]. According to Antonio-Nkondjio et al. [3], the Mount Cameroon Region is part of a holoendemic stratum with a strong and perennial malaria parasite transmission. Despite intensified efforts to reduce the burden of malaria in children less than 5 years old (including free bed net and malaria treatment by the government of Cameroon), the report of Danwang et al. [2] showed an upward trend in malaria incidence. Therefore, community surveys on the current burden of malaria and comorbidities such as anaemia, stunting and anisocytosis in this vulnerable age group will be very useful for a planned strategic control effort.

The aetiology of anaemia is often complex, and in African children, its epidemiology overlaps with a combination of nutritional deficiencies, infectious diseases (malaria and human immunodeficiency virus infections) [4, 5], helminth infections and the genetic constitution of red cell haemoglobin [6–8]. In Sub-Saharan countries particularly Cameroon, malaria is one of the key contributors to the public health problem of childhood anaemia [9–11], with *Plasmodium falciparum* causing the most severe anaemia, and a significant risk of death [9]. Anaemia can also occur in African children with apparently asymptomatic infections [12–14] however, in a high transmission setting, malaria raises the risk of anaemia in the entire population, but it has the largest impact on children under the age of five [15]. It is worth noting that childhood anaemia is a preventable disorder with major effects such as growth retardation, a weakened immune system, and greater susceptibility to diseases [16], and death [17] and has severe socio-economic consequences for families and communities. It is therefore important to determine its prevalence and severity in order to plan management strategies against the condition.

Indicators of blood such as haemoglobin level, mean cell corpuscular volume (MCV) and red blood cell distribution width (RDW) are used to analyse haematological changes. Low haemoglobin level reflects the severity of anaemia, meanwhile MCV and RDW are sensitive and specific indices to identify iron deficiency anaemia [18–20]. Low haemoglobin level along with a high level of anisocytosis as measured by red cell distribution width prove to be good markers of blood abnormalities caused by low iron storage [20, 21]. Therefore, there is a need to determine the burden of anaemia and anisocytosis as well as malnutrition among apparently healthy children in a malaria perennial transmission setting like the Mount Cameroon area.

Malnutrition is a complicated condition with a multiple aetiology and a wide range of clinical manifestations. Acute malnutrition is characterized by wasting (low weight for height), while chronic malnutrition is characterized by stunting (low height for age). In 2020, nearly 45% of deaths among children under the age of five were due to malnutrition with low and middle-income countries being the most affected [22]. Still in 2020, it was estimated that 149.2 million children under the age of five were stunted and 45.4 million were wasted globally [22]. Childhood is a time when key developmental milestones necessitate increased nutritional demands [23] and with the ongoing civil strife in the South West Region of Cameroon with many displaced especially mothers and children, the consequences are enormous [24], further increasing the vulnerability of these children. This therefore suggests a need for epidemiological data specific to this conflict zone, to assist decision-making in developing effective control programmes. Against this background, the study was aimed at determining the prevalence and severity of malnutrition, anaemia and anisocytosis as public health problems in children under five years, living in a perennial malaria transmission zone within the conflict hit Mount Cameroon area.

## Materials and methods

### Study area and participants

The study was carried out in 3 rural communities (Batoke, Tole and Dibanda) located in the Mount Cameroon area, Fako Division of the South West Region of Cameroon. These communities have been effectively described by Sumbele et al. [14]. The Mount Cameroon area has an equatorial climate characterized by abundant rainfall which varies from 1500 mm/year inland to 4000 mm/year on the seacoast and constant humidity [3]. This region is considered meso-hyperendemic with a high malaria parasite perennial transmission. Since 2017, these communities have experienced a civil strife between government forces and the armed separatist group following the Anglophone crisis in the English-speaking regions of Cameroon.

The study population included pre-school children of both sexes aged ≤ 5 years whose parent/caregiver consented through signing an informed consent form. This study included children who weighed more than 5 kg and excluded children with severe malaria, children who had blood transfused two months prior to the commencement of the study or had other diseases requiring immediate hospitalization.

### Study design, sampling technique and unit

The study design was cross-sectional and it was carried out in the three communities in Mount Cameroon area between April and May 2018. Each community was divided into blocks. Within each block, all the households with children ≤ 5 years of age were selected. In a household where only one child within that age was present, the child was selected automatically. In cases where more than one child within the age group was present in a household, only one was randomly selected. Dates for sample collection was scheduled for each selected family. Each head/leader of a block (quarter head) served as a relay agent to remind potential participants of their scheduled dates of sample collection. Participants were invited to the community’s sample collection location by the local chief and coordinated by head of the block.

Study methodology and benefits were explained to the parents and legal guardians. The study team proceeded with the collection of samples at specified identified collection sites after obtaining informed consent from the parents/care givers.

### Sample size and population

The sample size was calculated using the following formula [25]: *n* = *Z*^2^pq/*d*^2^; Where *z* = 1.96: confidence level test statistics at the desired level of significance; *p* = 32.6%, prevalence of malnutrition in children ≤ 5 years in the study area [26]; *q* = 1-*p*: proportion of healthy children and *d* = acceptable error willing to be committed (0.05). The minimum estimated sample size calculated was approximately 345. Assuming 15% non-response rate, the minimum sample size obtained was 397. To obtain a more insightful information, lesser margin of error, higher confidence level and models with more accuracy, the final sample size was increased to 628 individuals.

### Data collection

Responses on (i) demographics (gender, age, literacy level, occupation, and marital status); (ii) socioeconomic status-related variables (number of house occupants, house type, toilet type, and water sources); (iii) malaria knowledge level (signs/symptoms, complications, transmission, and prevention methods); (iv) Fever management methods; (v) malaria preventive practices, such as bed net ownership, physical integrity, number, and use); (vi) and indoor residual spray (IRS) experience were collected using a structured questionnaire adopted from Asoba et al. (2019) [26]. The use of long-lasting insecticide net was defined as sleeping under one the night before the survey [27].

### Clinical assessment

A digital thermometer was used to take the axillary temperature, and fever was defined as a temperature of 37.5^o^ C or higher. A measuring tape and a Terraillon weighing scale (Terraillon, Paris) were used to measure height and weight respectively. The tape was fastened to a locally produced woodwork that functioned as a stadiometer to ensure the precision. Malnutrition indices such as height-for-age (HA), weight-for-age (WA), and weight-for-height (WH) standard deviation (SD) scores (Z scores) were calculated using the WHO AnthroPlus for personal computers manual and the WHO growth reference curves [28]. If a child scored -2 on one of the anthropometric indicators, he or she was considered malnourished, whereas comparable Z scores of -3 SD were considered symptomatic of severe malnutrition.

### Detection and evaluation of malaria parasites

About 3–6 µL of whole blood were dispensed directly on the same slide for the preparation of thick and thin blood films, respectively, from the 4 mL of blood taken by venepuncture. The films were stained with 10% Giemsa and examined in the laboratory according to conventional methods [29]. Counting the number of parasites per 200 leukocytes on thick blood film and multiplying the parasite count with the participants' white blood cell count obtained from the complete blood count analysis yielded the malaria parasite density. Low parasite density (< 1000 parasites/µL blood), moderate parasite density (1000–4999 parasites/µL blood), high parasite density (5000–99,999 parasites/µL blood), and hyper parasitaemia (≥ 100,000 parasites/µL blood) were used to classify malaria parasite density [30].

### Determination of haematological parameters

Briefly, the blood samples were placed on a multi-mixer rotator for uniform mixing. A complete blood count was run following the manufacturer’s instructions using an auto-haematology analyser (MINRAY 2800 BC) to obtain haemoglobin concentration (Hb), haematocrit (Hct), mean corpuscular volume (MCV), mean corpuscular haemoglobin concentration (MCHC), mean corpuscular haemoglobin (MCH), and red blood cell distribution width coefficient of variation (RDW-CV).

### Definition of endpoints

Anisocytosis (RDW > 15%) [31].

Anaemia (Hb < 11 g/dL) and further classified as mild (Hb, 10.1–10.9 g/dL), moderate (Hb, 7.0–10.0 g/dL) and severe (Hb < 7 g/dL).

Microcytosis (MCV < 67 fL) for children < 2 years and MCV < 73 fL for children 2–5 years.

Microcytic anaemia (Hb < 11.0 g/dL + MCV < 67 fL or Hb < 11.0 g/dL + MCV < 73 fL) depending on the age [11].

WHO [32] classification of the prevalence of anaemia as a public health significance as: normal (prevalence of ≤ 4.9%; mild (prevalence of 5.0–19.9%); moderate (prevalence of 20.0–39.9%) and severe (prevalence of ≥ 40.0%).

### Statistical analysis

Data were entered into logbooks and then transferred to Microsoft Excel spreadsheets. The Statistical Package for Social Sciences (SPSS) version 23 (IBM-SPSS, Inc, Chicago, IL, USA) software was used to analyse the data after it was cleaned. The descriptive statistics were evaluated using percentages and haemoglobin levels were summarized into means and standard deviations (SD). The Chi-square test (χ2) was used to examine the comparison between malaria parasite, anaemia, anisocytosis and malnutrition (stunting, wasting and underweight) as dependent variables with demographic and clinical characteristics as independent variables. Prior to analysis, the malaria parasite densities were log transformed. To assess the intensity of infection in the study population, the geometric mean parasite densities (GMPDs) were employed, and differences were analysed using the Mann–Whitney *U* test and the Kruskal–Wallis test. Associations between predictor variables and anisocytosis were assessed using both bivariate and multivariate logistic regression analysis. Odd ratios (ORs) and 95% confidence intervals (CIs) were computed. Any covariate with a *P* value < 0.2 in the bivariate analysis was subsequently included in the final multivariable logistic model. Significant levels were measured at 95% confidence interval (CI) with significant differences designated at *P* < 0.05.

### Administrative approval and ethical considerations

Following an ethical clearance from the Institutional Review Board hosted by the Faculty of Health Sciences, University of Buea (2017/004/UB/FHS/IRB), the study was accepted with an administrative authorization from the South West Regional Delegation of Public Health, Cameroon. At presentation, informed consent/assent forms were given or read and explained to parents or caregivers of the children. The information page and consent/assent forms both clearly indicated the study's goal and advantages, as well as the amount of blood to be obtained from each child. The study included only children who gave verbal assent in addition to their parents written informed consent. Participation was entirely optional, and parents or caregivers had the right to withdraw their child from the study at any time. All the information acquired was kept in strict confidence. Although the data was coded, the identity of the sample that was analysed was not revealed. Malaria cases, as well as those with moderate-to-severe anaemia and malnutrition, were all referred to the nearest health facility for treatment and follow-up.

## Results

### Characteristics of participants and *Plasmodium* infection prevalence

A total of 628 preschool children of both sexes were included in the study. The overall prevalence of *P. falciparum* infection in the study area was 36.0% (226/628). Out of the 226 children positive for malaria, 33.3% were males and 38.4% females, with the ≤ 24 months age group having the highest prevalence of malaria when compared with the other age groups (Table 1). Children whose parents/caregivers had no formal level of education had the highest prevalence of *P. falciparum* infection (58.2%, 95% CI = 46.3–69.3), while those whose parents had a tertiary level of education had the least prevalence (19.2%, 95% CI = 11.8–29.7) at *P* < 0.001. In addition, the prevalence of *P. falciparum* infection was lower in children who owned a bed net (32.3%, 95% CI = 28.2–36.6) when compared with those who did not (47.4%, 95% CI = 39.7–55.3) at *P* < 0.001. Moreover, children who used bed nets (29.3%, 95% CI = 24.9–34.1) had a lower prevalence of malaria parasitaemia compared with those who didn’t (45.6%, 95% CI = 39.6–51.6) at *P* < 0.001. However, the overall prevalence of *P. falciparum* infection was similar among children with malnutrition, wasting, underweight and stunting when compared to those who did not. In contrast, prevalence of *P. falciparum* infection was significantly higher among children with anaemia (40.0%, 95% CI = 35.7–44.6) and anisocytosis (41.2%, 95% CI = 36.1–46.5) compared with those without anaemia (*P* < 0.001) and anisocytosis (*P* = 0.003).

**Table 1:** Descriptive characteristics by malaria parasitaemia at baseline

Variable	Category	No. examined	Malaria parasite prevalence % (*n*)	No. malaria parasite prevalence % (*n*)	*χ*^2^; *P* value
Sex	Male	300	33.3 (100)	66.7 (200)	1.756; 0.185
	Female	328	38.4 (126)	61.6 (202)	
Age group in months	≤ 24	224	40.2 (90)	59.8 (134)	4.233; 0.120
	25–47	145	29.7 (43)	70.3 (102)	
	48–60	259	35.9 (93)	64.1 (166)	
Educational level of parent/caregiver	No formal	67	58.2 (39)	41.8 (28)	25.615; < 0.001***^a^
	Primary	236	32.2 (76)	67.8 (160)	
	Secondary	197	39.1 (77)	60.9 (120)	
	Tertiary	73	19.2 (14)	80.8 (59)	
Marital status of parent/caregiver	Single	143	31.5 (45)	68.5 (98)	2.610; 0.106^b^
	Married	394	39.1 (154)	60.9 (240)	
ITN ownership	Yes	474	32.3 (153)	67.7 (321)	11.542; < 0.001***
	No	154	47.4 (73)	52.6 (81)	
Use of ITN	Yes	369	29.3 (108)	70.7 (261)	17.534; < 0.001***
	No	259	45.6 (118)	54.4 (141)	
Fever	Yes	54	48.1 (26)	51.9 (28)	3.793; 0.051
	No	574	34.8 (200)	65.2 (374)	
Malnourished	Yes	257	38.9 (100)	61.1 (157)	1.614; 0.204
	No	371	34.0 (126)	66.0 (245)	
Wasted	Yes	114	40.4 (46)	59.6 (68)	1.151; 0.283
	No	514	35.0 (180)	65.0 (334)	
Underweight	Yes	133	39.1 (52)	60.9 (81)	0.709; 0.400
	No	495	35.2 (174)	64.8 (321)	
Stunted	Yes	182	40.7 (74)	59.3 (108)	2.428; 0.119
	No	446	34.1 (152)	65.9 (294)	
Anaemia	No	171	25.1 (43)	74.9 (128)	11.989; < 0.001***
	Yes	457	40.0 (183)	60.0 (274)	
Microcytosis	Yes	270	38.1 (103)	61.9 (167)	0.960; 0.327
	No	358	34.4 (123)	65.6 (235)	
Anisocytosis	Yes	340	41.2 (140)	58.8 (200)	8.666; 0.003**
	No	288	29.9 (86)	70.1 (202)	

^a^Educational level of parent/caregiver was evaluated for 573 participants

^b^Marital status of parent/caregiver was evaluated for 537 participant

**Statistically significant at *P* < 0.01; ***Statistically significant at *P* < 0.001

### Malnutrition and its categories in relation to age, sex and malaria parasitaemia

**Fig. 1:**
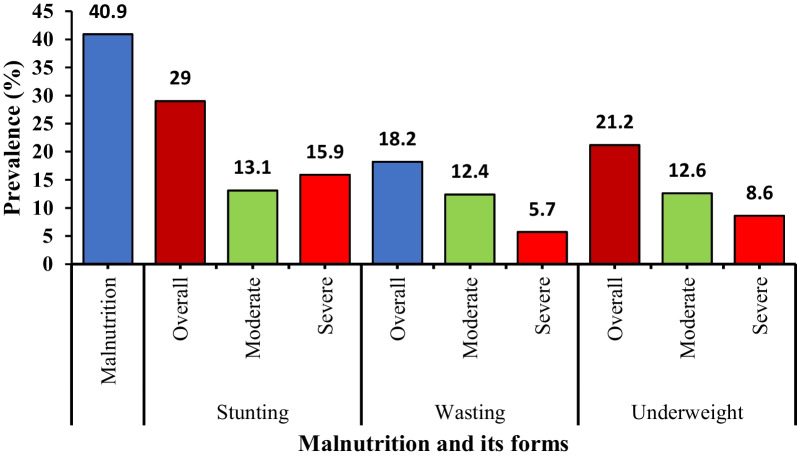
The prevalence of malnutrition and its severity in the study population

The overall prevalence of malnutrition, stunting, wasting and underweight in the study population was 40.9% (95% CI = 37.1–44.8), 29.0% (95% CI = 25.6–32.6), 18.2% (95% CI = 15.3–21.4), and 21.2% (95% CI = 18.2–24.5), respectively (Fig. 1). Most of the children were moderately wasted (12.4%) and underweight (12.6%) when compared with the severe forms. However, 15.9% of the children were severely stunted while 13.1% were moderately stunted.

**Fig. 2:**
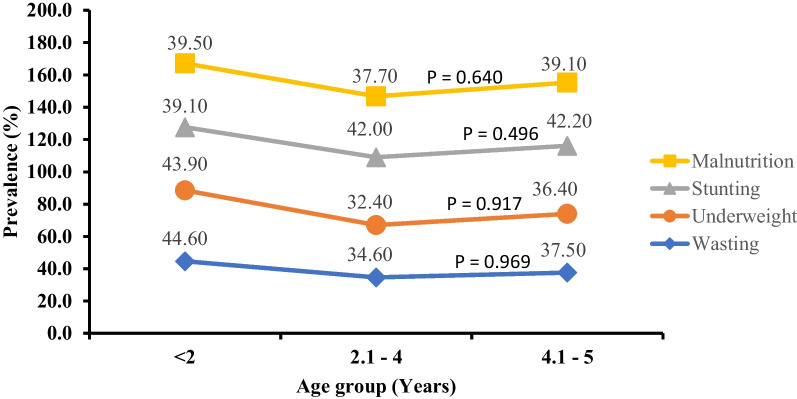
Prevalence of malnutrition and its categories with respect to age

**Fig. 3:**
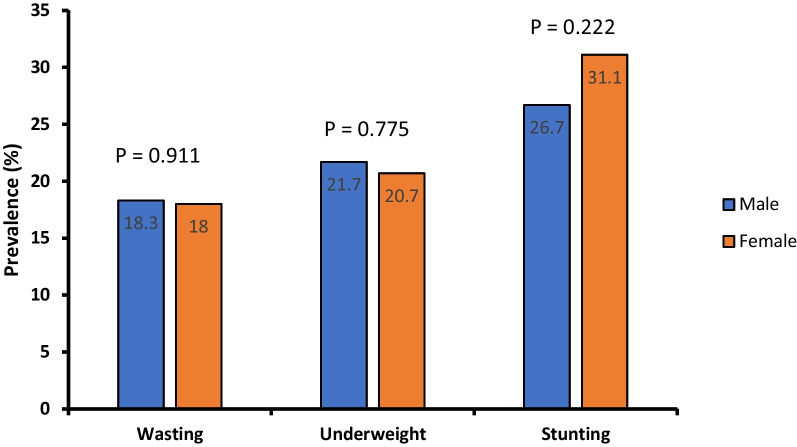
Prevalence of wasting, underweight and stunting with respect to sex

As seen in Fig. 2, children in the ≤ 24 months age group were the most malnourished, wasted and underweight when compared with their age counterparts although the differences were not statistically significant. Similarly, sex was not significantly associated with either wasting, underweight, or stunting (Fig. 3).

As seen in Table 2, the overall prevalence of moderate and severe wasting were 12.4 (95% CI = 10.1–15.2) and 5.7% (95% CI = 4.2–7.3), respectively, with no significant differences in sex observed. In like manner, 12.6% (95% CI = 10.2–15.4) and 8.6% (95% CI = 6.6–11.1) of the children were moderately and severely underweight while moderate and severe stunting was prevalent in 13.1% (95% CI = 10.6–15.9) and 15.9% (95% CI = 13.3–19.0) of the children, respectively, with no significant difference in sex.

**Table 2:** Prevalence of wasting, underweight, stunting and their severity with respect to sex and age

Variable	No. examined	Wasting		Underweight		Stunting	
		Moderate% (*n*)	Severe % (*n*)	Moderate % (*n*)	Severe% (*n*)	Moderate% (*n*)	Severe% (*n*)
Sex							
Male	300	12.3 (37)	6.0 (18)	11.7 (35)	10.0 (30)	12. 7(38)	14.0 (42)
Female	328	12.5 (41)	5.5 (18)	13.4 (44)	7.3 (24)	13.4 (44)	17.7 (58)
Total	628	12.4 (78)	5.7 (36)	12.6 (79)	8.6 (54)	13.1 (82)	15.9 (100)
* χ*^2^; *P* value		0.077; 0.962		1.710; 0.425		1.835; 0.400	
Age group/ months							
≤ 24	224	17.4 (39)	7.6 (17)	18.8 (42)	10.7 (10.7)	14.7 (33)	24.1 (54)
25–47	265	13.6 (36)	3.8 (10)	12.8 (34)	8.7 (23)	13.2 (35)	13.6 (36)
48–60	139	2.2 (3)	6.5 (9)	2.2 (3)	5.0 (7)	10.1 (14)	7.2 (10)
Total	628	12.4 (78)	5.7 (36)	12.6 (79)	8.6 (54)	13.1 (82)	15.9 (100)
*χ*^2^; *P* value		22.664; < 0.001***		27.416; < 0.001***		24.348; < 0.001***	

***Statistically significant at *P* < 0.001

Of statistical significance, the ≤ 24 months age group were more moderately, wasted (17.4%, 95% CI = 13.0–22.9), underweight (18.8%, 95% CI = 14.2–24.4) and stunted (14.7%, 95% CI = 10.7–20.0) than their older counterparts at *P* < 0.001, *P* < 0.001 and *P* < 0.001, respectively, (Table 2).

**Fig. 4:**
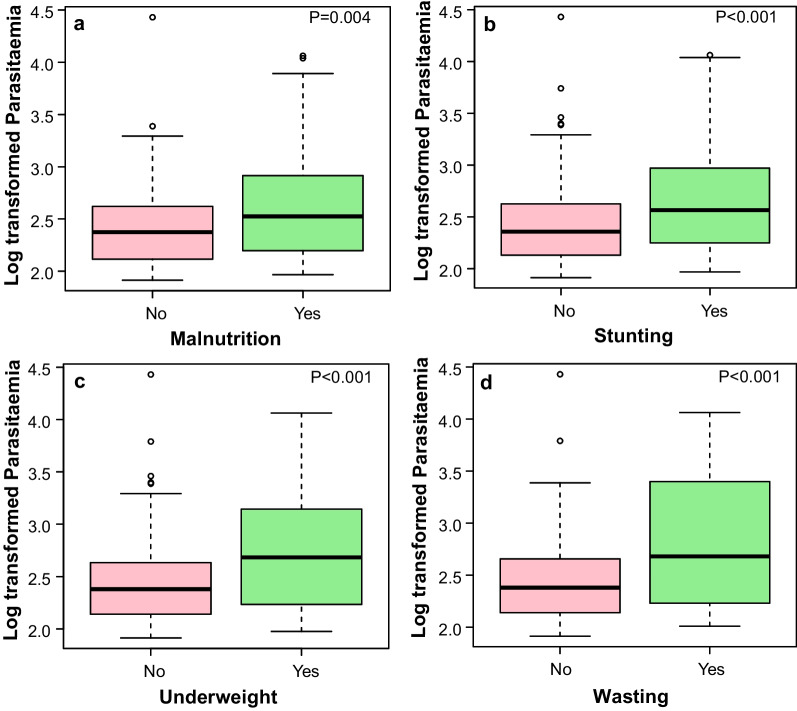
Mean log transformed parasite density as affected by **a** malnutrition, **b** stunting, **c** underweight, and **d** wasting

As seen in Fig. 4, the mean log malaria parasites/µL of blood was significantly higher among malnourished (2.59, 95% CI = 2.52–2.66), stunted (2.63, 95% CI = 2.55–2.71), wasted (2.73, 95% CI = 2.61–2.85) and underweight (2.71, 95% CI = 2.61–2.81) children when compared with their well-nourished counterparts at *P* = 0.004, *P* < 0.001, *P* < 0.001 and *P* < 0.001, respectively.

### Influence of socio-demographic and clinical factors on anaemia and anisocytosis

The mean Hb level in the study population was 10.04 g/dL with children ≤ 24 months having the least mean haemoglobin level (9.69 g/dL, 95% CI = 9.4–9.9) when compared with their older counterparts at *P* < 0.001, as shown in Table 3. Malnourished children had a similar mean haemoglobin level when compared with the well-nourished children. However, stunted children had a lower haemoglobin level (9.74 g/dL, 95% CI = 9.5–10.01) when compared with their non-stunted counterparts (10.16 g/dL, 95% CI = 10.0–10.3) at *P* = 0.014.

**Table 3:** Mean haemoglobin levels (g/dL) as affected by demographic, malnutrition and it forms

Variable	*N*	Mean Hb in g/dL	SD	95% CI for mean	*t* test
					*P* value
Sex					
Male	300	10.04	1.99	9.8–10.3	0.014
Female	328	10.04	1.87	9.8–10.2	0.989
Age group/ months					
< 24	224	9.69	1.85	9.4–9.9	6.613^a^
25–47	265	10.14	2.11	9.9–10.4	
48–60	139	10.40	1.56	10.2–10.6	0.001***
Malnourished					
Yes	257	10.01	1.96	9.8–10.2	− 0.348
No	371	10.06	1.90	9.9–10.3	0.728
Stunted					
Yes	182	9.74	1.81	9.5–10.01	− 2.464
No	446	10.16	1.96	10.0–10.3	0.014*
Underweight					
Yes	133	10.24	1.99	9.9–10.6	1.350
No	495	9.98	1.90	9.8–10.2	0.177
Wasted					
Yes	114	10.41	2.08	10.0–10.8	1.261
No	514	10.01	1.88	9.8–10.1	0.064

^a^Means compared using *F* test, *Statistically significant at *P* < 0.05, ***Statistically significant at *P* < 0.001

Anaemia, microcytic anaemia, non-microcytic anaemia and anisocytosis was prevalent in 72.8% (95% CI = 69.2–76.1), 30.1% (95% CI = 26.6–33.8), 42.7% (95% CI = 38.9–46.7) and 54.1% (95% CI = 50.2–58.0) of the study population, respectively. Sex did not significantly affect the prevalence of anaemia, microcytic anaemia and anisocytosis as shown in Table 4. Children ≤ 24 months old did not have a significantly higher prevalence of anaemia than their counterparts while, the highest prevalence of microcytic anaemia (38.8%, 95% CI = 32.7 – 45.4) and anisocytosis (68.8%, 95% CI = 62.4 – 74.5) was observed in this age group when compared with the older children at *P* < 0.002 and *P* < 0.001, respectively.

**Table 4:** Prevalence of anaemia, microcytic anaemia, non-microcytic anaemia and anisocytosis with respect to socio-demographic and clinical factors

Parameter	Category	*N*	Prevalence [% (*n*)] of			
			Anaemia	Microcytic anaemia	Non-microcytic anaemia	Anisocytosis
Sex	Male	300	71.7 (215)	30.0 (90)	41.7 (125)	52.0 (156)
	Female	328	73.8 (242)	30.2 (99)	43.6 (143)	56.1 (184)
*P* value			0.552	0.820		0.303
Age group in months	< 24	224	76.3 (171)	38.8(87)	37.5 (84)	68.8 (154)
	25–47	265	71.7 (190)	28.7 (76)	43.0 (114)	52.8 (140)
	48–60	139	69.1 (96)	18.7 (26)	50.4 (70)	33.1 (46)
*P* value			0.278	0.002**		< 0.001***
Educational level of parent/caregiver	No formal	67	83.6 (56)	31.3 (21)	52.2 (35)	23.9 (16)
	Primary	236	72.0 (170)	30.5 (72)	41.5 (98)	63.6 (150)
	Secondary	197	76.6(151)	37.1 (73)	39.6 (78)	62.4 (123)
	Tertiary	73	78.1(57)	21.9 (16)	56.2 (41)	38.4 (28)
*P* value			0.230	0.061		< 0.001***
Fever	No	574	72.6 (417)	30.0 (172)	42.7 (245)	53.0 (304)
	Yes	54	74.1 (40)	31.5 (17)	42.6 (23)	66.7 (36)
*P* value			0.822	0.963		0.053
Malnourished	Yes	257	70.8 (182)	33.1 (85)	37.7 (97)	70.0 (180)
	No	371	74.1 (275)	28.0 (104)	46.1 (171)	43.1 (160)
*P* value			0.360	0.112		< 0.001***
Wasted	Yes	114	64.9 (74)	30.7 (35)	34.2 (39)	78.9 (90)
	No	514	74.5 (383)	30.0 (1540	44.6 (229)	48.6 (250)
*P* value			0.037*	0.063		< 0.001***
Underweight	Yes	133	66.9 (89)	28.6 (38)	38.3(51)	76.7 (102)
	No	495	74.3 (368)	30.5 (151)	43.8 (217)	48.1 (238)
*P* value			0.088	0.224		< 0.001***
Stunted	Yes	182	74.7 (136)	33.5 (61)	41.2 (75)	65.9 (120)
	No	446	72.0 (321)	28.7 (128)	43.3 (193)	49.3 (220)
Total		628	72.8 (457)	30.1 (189)	42.7 (268)	54.1 (340)
*P* value			0.482	0.476		< 0.001***

*Statistically significant at *P* < 0.05, **Statistically significant at *P* < 0.01, ***Statistically significant at *P* < 0.001

Furthermore, the prevalence of anaemia and microcytic anaemia was similar between the well-nourished and malnourished, wasted, underweight and stunted children. Nevertheless, the prevalence of anisocytosis was higher among malnourished (70.0%), wasted (78.9%), underweight (76.7%), stunted (65.9%) when compared with their well-nourished counterparts at *P* < 0.001 (Table 4).

### Risk factors of anisocytosis

The logistic regression model with anisocytosis as dependent variable and sex, age, family size, educational level of parents/caregivers, fever, malnutrition, stunting, wasting, underweight, malaria, as well as malnutrition demonstrated that children who were malnourished (*P* = 0.005), had malaria parasite (*P* = 0.007), and whose parents had a primary (*P* = 0.002) and secondary levels of education (*P* = 0.017) were more likely to have anisocytosis. Children from parents with primary and secondary level of education were 3.51 and 2.69 times at higher odds of having anisocytosis than children from parents with no formal level of education, as shown in Table 5. Also, children with malnutrition and malaria, were 4.66 and 1.87 times, respectively, more likely to have anisocytosis than their counterparts.

**Table 5:** Logistic regression model examining factors associated with anisocytosis in the study population

Variables	*N*	Bivariate logistic regression		Multivariate logistic regression	
		COR (95% CI)	*P* value	AOR	*P* value
Sex					
Male	300	Reference		Reference	
Female	328	1.18 (0.86–1.62)	0.303	1.13 (0.75–1.69)	0.572
Age group (years)					
< 24	224	2.26 (1.48–3.47)	0.001**	1.22 (0.67–2.19)	0.512
25–47	265	4.45 (2.83–6.99)	< 0.001***	1.73 (0.94–3.18)	0.081
48–60	139	Reference		Reference	
Educational level of parent/caregiver					
No formal	67	Reference		Reference	
Primary	236	5.56 (2.99–10.35)	< 0.001***	3.51 (1.58–7.81)	0.002**
Secondary	197	5.30 (2.82–9.96)	< 0.001***	2.69 (1.20–6.04)	0.017*
Tertiary	73	1.98 (0.95–4.13)	0.067	1.34 (0.46–3.84)	0.591
Fever					
No	574	Reference		Reference	
Yes	54	1.78 (0.99–3.20)	0.056	1.89 (0.85–4.17)	0.117
Malnourished					
No	371	Reference		Reference	
Yes	257	3.08 (2.20–4.32)	< 0.001***	4.66 (1.56–13.92)	0.005**
Stunting					
No	446	Reference		Reference	
Yes	182	1.99 (1.34–2.85)	< 0.001***	0.44 (0.16–1.23)	0.117
Wasted					
No	514	Reference		Reference	
Yes	114	3.96 (2.44–6.41)	< 0.001***	1.56 (0.58 (4.21)	0.379
Underweight					
No	495	Reference		Reference	
Yes	133	3.55 (2.29–5.51)	< 0.001***	1.27 (0.57–2.83)	0.564
Malaria					
No	402	Reference		Reference	
Yes	226	1.64 (1.18–2.29)	0.003**	1.85 (1.18–2.91)	0.007**
Anaemia					
No	171	Reference		Reference	
Yes	457	1.24 (0.87–1.76)	0.237	1.35 (0.84–2.18)	0.217

*Statistically significant at P < 0.05, **Statistically significant at P < 0.01, ***Statistically significant at P < 0.001

## Discussion

Childhood is a time when the major developmental milestones are more nutritionally demanding and could be compromised by malaria, anaemia, anisocytosis and malnutrition which are serious public health concerns, particularly in low- and middle-income nations such as Cameroon.

The overall malaria parasitaemia of 36.0% observed by microscopy in the study population confirms earlier studies by Asoba et al. [26] that malaria remains a major cause of illness during childhood and is still meso endemic in this part of the Mount Cameroon area. This frequency was comparable to the 35.3% reported by Eyong et al. [33] in children under the age of five in other parts of Mount Cameroon. However, Tabue et al. [34] reported that parasite prevalence among this age group ranged from 15.48% in Myo Olu to 24.6% in Garoua and 45.6% in Pitoa in Cameroon's North Region, where malaria transmission is seasonal. Furthermore, the malaria prevalence in this age range was higher than the 15.94% observed in under five-year-olds in southern Tanzania, where malaria transmission was largely seasonal [35]. The greater malaria prevalence seen in this study could be due to the equatorial climate of Mount Cameroon, which is characterized by plentiful rainfall and persistent humidity, both of which are variables that favour strong and perpetual malaria transmission [9].

According to the findings, children who owned and used their ITNs had a considerably decreased prevalence of malaria parasitaemia. This is consistent with the findings of Yekabong et al*.* [36] in Cameroon's South-West Region, as well as other South American investigations [37].

In line with the findings of Ebai et al. [38] in other parts of the Mount Cameroon area, children from individuals with no formal or with primary education were more infected with the malaria parasite than those with secondary or tertiary education. As reported by several authors [39, 40], higher levels of education are linked to better understanding and practices when it comes to effective preventative and treatment techniques.

When compared with their peers, malaria prevalence was greater in anaemic (40.0%) children and those with anisocytosis (41.2%). In addition, findings from the study indicated malaria positive children were 1.87-fold more likely have anisocytosis, when compared with their negative counterparts. In previous investigations, the link between malaria parasitaemia and anaemia has been clearly established [9, 11]. Malaria parasitaemia causes more parasitized and non-parasitic red blood cells to be destroyed, lowering haemoglobin levels and resulting in anaemia. However, the link between anisocytosis and malaria could indicate that the youngsters had iron deficiency anaemia. Akkermans et al. [41] reported that red blood cell distribution width (RDW) which is a measure of anisocytosis may be helpful for identifying iron deficiency (ID) as cause of anaemia in young children.

The burden of malnutrition (40.9%), stunting (29.0%), wasting (18.2%), and underweight (21.2%) in the population represents serious public health concern in the Mount Cameroon area. These observations matched those of Manjong et al. [42] who reported a 55.08% stunting, 13.77% wasting and 31.99% underweight among under-five indigenous Mbororo children in another community in Cameroon. From 1990 to 2014, the prevalence of stunting, wasting, and underweight in Cameroon increased from 24.4% to 32%, 3% to 5.2%, and 13.6% to 14.8%, respectively, according to UNICEF [43]. This progressive rise in the burden of undernutrition in Cameroon is a cause for concern, particularly in this context, when the morbidity is aggravated by ongoing civil violence.

The burden of malnutrition, stunting, wasting and underweight in the ≤ 24 months were comparable to that of their older counterparts. However, the ≤ 24 months were more moderately wasted (17.4%), underweight (18.8%) and stunted (14.7%) than their older age counterparts. Previous research has linked the onset of growth stalling with an increased chance of death by the age of 24 months [44]. Also, according to a review by Thurstans et al. [45], the peak age of wasting and stunting is from birth to three months, with implications for subsequent deterioration in infancy and childhood. In a multi-country longitudinal analysis, Mertens et al. [46] found that chronic wasting from birth to 6 months (defined as > 50% of measures wasted) was highly linked with incident stunting at older ages.

Malaria and undernutrition morbidity are frequently linked, and children who are seriously afflicted by one are frequently afflicted by the other. Malnourished, stunted, wasting, and underweight children had considerably greater malaria parasitaemia. This is in line with a study from Ethiopia that reported malaria as a risk factor for malnutrition [47]. Undernutrition has long been recognized as both a cause and a consequence of infectious illness probably through decreased or changed nutritional intake, impaired intestinal absorption, and increased metabolism caused by fever, immunological response, and environmental enteropathy. [48].

The prevalence of anaemia (72.8%), microcytic anaemia (30.1%), and anisocytosis (54.1%), among the children in this study area, is still unacceptably high. This study's anaemic prevalence is similar to the 77.3% found by Asoba et al. [26] among 5-year-olds in other Mount Cameroon communities. Microcytic anaemia was less common than the 43.3% reported by Sama et al.[49] among urban children aged ≤ 5 years in the Mount Cameroon region. Furthermore, as compared to their older counterparts, the ≤ 24 months-olds had a greater prevalence of microcytic anaemia (38.8%) and anisocytosis (68.8%), which could be indicative of iron deficiency anaemia. Findings from this study indicated malnourished children were 4.66-fold more likely to have anisocytosis, when compared with their negative counterparts. Furthermore, when compared to their well-nourished counterparts, children who were wasted (78.9%), underweight (76.7%), and stunted (65.9%) showed a higher prevalence of anisocytosis. Increased RDW reflects a substantial dysregulation of erythrocyte homeostasis, which includes both defective erythropoiesis and aberrant red blood cell survival, and can be related to a range of underlying metabolic disorders, such as low nutritional status [50]. Due to insufficient iron supply, anisocytosis occurs, in which the erythrocytes generated are smaller than average in size and have a considerable size variance [41].

## Limitation

Anthropometric indices were used to determine nutritional status, which may have underestimated the magnitude when compared to biochemical data. It would have been beneficial to add the dietary diversity score and household food insecurity status, both of which have a causal relationship with children's nutritional status. Nevertheless, the high prevalence of undernutrition among the children in this study warrants immediate intervention.

## Conclusion

Malaria, anaemia, anisocytosis and undernutrition are still public health concerns among children ≤ 5 years in the Mount Cameroon area. Evidence on the cumulative negative impact of these comorbidities especially stunting is seen over a child's lifetime. It highlights the need for a more integrated approach for preventative and treatment techniques to stop this process. This therefore emphasizes the importance of consistent policy and the implementation of programmes and activities to avoid malaria, anaemia, anisocytosis and undernutrition particularly stunting in children under 60 years.

## Data Availability

All datasets on which the conclusions of the research rely are presented in this paper. However, data are available from the corresponding author on reasonable request.
